# Comparative effectiveness of dexamethasone versus methylprednisolone for neuropathic outcomes after combined spinal-epidural labor analgesia: a prospective, randomized, double-blind trial

**DOI:** 10.3389/fmed.2026.1785911

**Published:** 2026-03-25

**Authors:** Bo Liu, Xiaoyan Chen, Huiru Li, Na Du, Li Luo, Siyan Dou, Xiaoqin Tang, Na Li, Fei Jia

**Affiliations:** Department of Anesthesiology, Chengdu Jinjiang District Women & Children Health Hospital, Chengdu, Sichuan, China

**Keywords:** combined spinal-epidural, dexamethasone, labor analgesia, methylprednisolone, neurological complications

## Abstract

**Background:**

Paresthesias occurring during combined spinal-epidural labor analgesia due to needle puncture or epidural catheter placement may lead to nerve injury. We compared the effects of epidural dexamethasone versus methylprednisolone on neurological outcomes.

**Methods:**

In this prospective, randomized, double-blind clinical trial conducted at a Chinese obstetrics hospital, parturients who developed paresthesias during combined spinal-epidural labor analgesia due to needle puncture or epidural catheter placement were randomized to receive an epidural injection of dexamethasone (5 mg) or methylprednisolone (40 mg). The primary outcome was neurological function at 14 days postpartum, including impaired skin sensation, decreased muscle strength, or other neurological symptoms. Secondary outcomes included adverse events and changes in inflammatory biomarkers.

**Results:**

A total of 315 parturients were randomized and completed the study. At 14 days postpartum, the neurological function outcomes did not differ significantly between the two groups (*p* > 0.05). Among the secondary outcomes, the dexamethasone group had a higher incidence of epidural-related maternal fever (ERMF) (7.6% vs. 2.5%, *p* < 0.05), and the 24-h postpartum serum Interleukin-6 (IL-6) level was significantly higher than that in the methylprednisolone group [26.1 (18.2–38.9) pg./mL vs. 15.4 (13.2–17.6) pg./mL, *p* < 0.05]. There were no significant differences between the two groups in postpartum hemorrhage, nausea and vomiting, instrumental delivery rate, Neonatal Intensive Care Unit (NICU) admission rate, or 24-h postpartum serum C-reactive protein (CRP) level (*p* > 0.05).

**Conclusion:**

In parturients who developed paresthesia during combined spinal-epidural labor analgesia, epidural administration of dexamethasone or methylprednisolone resulted in comparable neurological outcomes at 14 days postpartum.

**Clinical trial registration:**

https://www.chictr.org.cn/, identifier ChiCTR2300078866

## 1 Introduction

During combined spinal-epidural anesthesia, puncture or epidural catheter placement frequently triggers paresthesias, suggesting that the needle tip or the epidural catheter may contact or compress a nerve. These paresthesias may be accompanied by transient neuralgia and radicular pain; if symptoms persist or worsen, neurological impairment may occur, including nerve root injury or cauda equina syndrome, and, rarely, permanent neurological damage (1–5). Combined spinal-epidural analgesia provides rapid onset and effective analgesia and is widely used for labor analgesia (6). However, during labor analgesia, maternal pain and tension can affect cooperation, which can make puncture more difficult and may increase the risk of neurological complications (7). Although paresthesia during labor analgesia is often transient and self-limiting, the potential for persistent neurological symptoms justifies exploration of prophylactic interventions. However, evidence supporting epidural corticosteroid use in this specific obstetric context remains limited.

Epidural administration of glucocorticoids inhibits inflammatory enzymes and cytokines, reduces local edema and inflammatory cell infiltration, improves the perineural environment, relieves pain, and promotes recovery of neurological function (8, 9). Dexamethasone is a non-particulate glucocorticoid, and the literature reports that its epidural injection has a certain effect in controlling symptoms related to nerve root injury, and may be associated with less immunosuppression and other adverse effects (10, 11). Methylprednisolone epidural injection likewise has anti-inflammatory effects in treating nerve injury (12, 13). However, no studies have directly compared these two drugs for neurological outcomes following puncture- or catheter-related paresthesia during combined spinal-epidural labor analgesia.

To address this gap, we designed a randomized clinical trial to directly compare the effects of epidural dexamethasone versus methylprednisolone on neurological outcomes in parturients who experience paresthesia during combined spinal-epidural labor analgesia.

## 2 Methods

### 2.1 Study design

We conducted a prospective, randomised, double-blind clinical trial at a large tertiary obstetric hospital in China, with an annual delivery volume of approximately 5,000. The study was approved by the Ethics Committee of Chengdu Jinjiang District Women & Children Health Hospital (approval no. 202313) and registered with the Chinese Clinical Trial Registry (registration no. ChiCTR2300078866; accessible at https://www.chictr.org.cn/). The study was conducted and reported in accordance with the Consolidated Standards of Reporting Trials (CONSORT) guidelines and adhered to the principles of the Declaration of Helsinki. Written informed consent was obtained from all patients.

### 2.2 Patients

Participant recruitment began in January 2024. Inclusion criteria: American Society of Anesthesiologists (ASA) physical status II; age ≥ 18 years; primipara; singleton full-term pregnancy; Body mass index (BMI) ≤ 35 kg/m^2^; baseline absence of back or leg neurological symptoms; voluntary acceptance of combined spinal–epidural labor analgesia; and the occurrence of paresthesias during puncture or epidural catheter placement (as a final inclusion criterion). Exclusion criteria: contraindications to neuraxial analgesia; allergy to corticosteroids; severe hepatic or renal impairment or other organ dysfunction; history of neuraxial anesthesia; conversion to cesarean delivery; or refusal to sign informed consent.

### 2.3 Randomization and blinding

#### 2.3.1 Randomization

Participants were randomly assigned in a 1:1 ratio to the dexamethasone group (Group D) or the methylprednisolone group (Group M) using block randomization with a fixed block size of 6. The random sequence was computer-generated in advance by an independent statistician not involved in trial implementation. Upon confirmation of paresthesia, the coordinator opened the next envelope to determine group allocation, which was then used by the independent pharmacy to prepare the corresponding blinded study medication. The attending anesthesiologist subsequently administered this pre-prepared, visually identical medication.

#### 2.3.2 Blinding

This was a double-blind trial. Participants, outcome assessors, and data analysts remained blinded to group assignment throughout the study. Study medications (dexamethasone or methylprednisolone) were prepared by an independent pharmacist not involved in clinical care or outcome assessment. Both drugs were diluted to a total volume of 5 mL with 0.9% normal saline, drawn into identical syringes, and affixed with an opaque white label bearing only the participant’s study identification number. All postpartum assessments, including neurological examinations and collection of inflammatory markers, were conducted by researchers unaware of group allocation. Data entry and statistical analysis were also performed by personnel blinded to treatment assignment.

Emergency unblinding was permitted only in cases of serious adverse events where clinical management required knowledge of the study drug, and such unblinding required approval from the principal investigator.

### 2.4 Combined spinal-epidural labor analgesia

All enrolled parturients presenting to the delivery room with cervical dilation ≥1 cm received combined spinal–epidural labor analgesia. Before labor analgesia, standard monitoring was performed, including electrocardiogram, SpO2, noninvasive blood pressure, respiratory rate and temperature. An upper-extremity venous access was established, and an intravenous infusion of compound sodium chloride solution was administered at 2–4 mL/kg/h. The parturients were placed in the lateral decubitus position, and the L3–L4 interspace was selected for puncture. Following successful epidural puncture, a pencil-point spinal needle was used to perform a subarachnoid puncture; after confirming free cerebrospinal fluid flow, 2 mL of a mixture of 0.1% ropivacaine and 0.5 μg/mL sufentanil was injected. Subsequently, an epidural catheter was advanced cephalad through the epidural needle to a depth of 3–4 cm. Through the epidural catheter, 3 mL of 1.5% lidocaine was administered as a test dose to exclude intravascular placement and to preliminarily confirm the blockade level.

### 2.5 Intervention

If a parturient experiences a definite electric-shock sensation or radiating pain in the lower limbs during subarachnoid puncture or epidural catheter placement, this meets the intervention criteria for this study. After the epidural catheter is secured and the trial dose is administered, the attending anesthesiologist should inform the study coordinator. The study coordinator opens the sealed randomization envelope to reveal the group assignment (Group D or Group M) and notifies the independent pharmacy staff to prepare the corresponding drugs. Per the notification, the pharmacy staff dilutes dexamethasone 5 mg or methylprednisolone 40 mg with 0.9% saline to a total volume of 5 mL and dispenses them into visually indistinguishable 5 mL syringes. The syringes are not labeled with the drug name; instead, an opaque label bearing the subject’s study number is affixed. The prepared syringes are then handed to the anesthesiologist. The anesthesiologist administers the premixed solution through the epidural catheter and, after injection, re-aspirates to confirm proper placement.

### 2.6 Labor analgesia management

Thirty to forty-five minutes after the trial drug injection, if no abnormalities are observed, the anesthesiologist should connect the epidural catheter to an electronic analgesia pump. The analgesia pump operates in a programmed epidural intermittent infusion mode. The drug formulation is 0.1% ropivacaine plus 0.5 μg/mL sufentanil, diluted with 0.9% saline to a total volume of 100 mL. Settings are as follows: bolus dose 8 mL, basal infusion rate 2 mL/h, patient-controlled dose 5 mL per request, maximum hourly dose 25 mL, and patient-controlled lockout interval 20 min. Obstetric ward staff and the anesthesiologist will jointly monitor analgesia efficacy, vital signs, and neurological status until delivery. Following delivery, the parturient was observed for a further 2 h before being transferred back to the postpartum ward.

### 2.7 Data collection

The primary outcome was the maternal neurological status at 14 days postpartum, including impaired skin sensation, decreased muscle strength, and other neurological symptoms.

Secondary outcomes included: maternal serum inflammatory markers (CRP and IL-6) measured within 24 h before labor analgesia and at 24 h postpartum; adverse events, including the incidence of ERMF (body temperature ≥38.0 °C) and nausea/vomiting; the rate of instrumental delivery; and the rate of neonatal admission to the NICU.

All parturients were followed up by telephone at 14 days postpartum to inquire about neurological outcomes. Blood samples for inflammatory markers were collected within 24 h before analgesia and at 24 h after delivery. Adverse events were monitored and recorded continuously during labor and throughout the hospital stay.

### 2.8 Statistical analysis

#### 2.8.1 Sample size calculation

The sample size was calculated using PASS 15.0 software. To date, no prospective studies have directly compared dexamethasone and methylprednisolone for neurological outcomes following paresthesia related to neuraxial procedures. Therefore, we referenced a report from a comparable clinical context involving steroid treatment for neurological injury or inflammation (14). In that study, the rates of significant neurological improvement were 27.3% with dexamethasone and 45.8% with methylprednisolone. Using this difference, with a power (1-*β*) of 90% and a two-sided *α* of 0.05, we estimated that 141 participants per group would be required. Allowing for a 10% loss to follow-up, we planned to enroll at least 314 participants (157 per group). Ultimately, 315 participants were included in the study.

#### 2.8.2 Statistical methods

Statistical analyses were performed via SPSS software version 25.0 and R4.3.1. The normality of data distribution was assessed using the Kolmogorov–Smirnov test. Normally distributed continuous variables are expressed as mean ± standard deviation (SD) and analyzed with independent-samples *t*-tests. Non-normally distributed continuous variables are expressed as median (interquartile range, IQR) and analyzed using the Mann–Whitney *U* test. Categorical variables are presented as numbers and percentages (%) and compared with the Chi-square test or Fisher’s exact test as appropriate. A two-tailed *p*-value < 0.05 was considered statistically significant.

## 3 Results

### 3.1 Parturient enrollment and baseline characteristics

**Figure 1:**
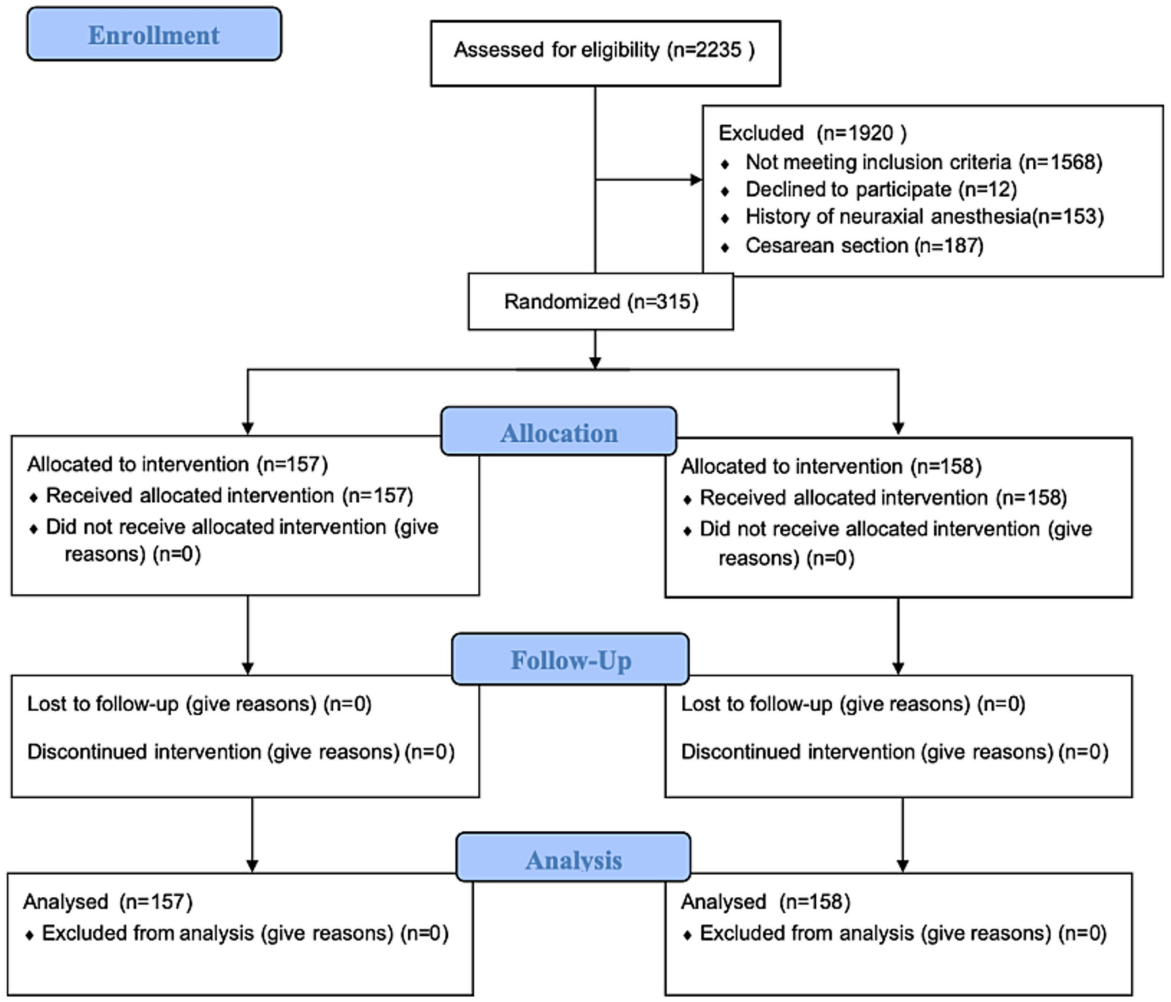
Flowchart of the patient selection and inclusion process.

From January 2024 to October 2025, a total of 2,235 parturients planning for labor analgesia were screened. Of these, 315 met the inclusion criteria and were randomly assigned in a 1:1 ratio to the D group (*n* = 157) or the M group (*n* = 158). All parturients received the allocated intervention, and there were no losses to follow-up during the follow-up period (Figure 1). Baseline demographic and clinical characteristics did not differ significantly between the two groups (Table 1).

**Table 1:** Baseline characteristics of the parturients.

Variable	Group D (*n* = 157)	Group M (*n* = 158)	*p-*value
Age (years)	29 (27–32)	28 (27–31)	0.120
BMI (Kg/m^2^)	26.1 (24.5–28.5)	26.0 (24.0–28.2)	0.297
GDM	31 (19.7%)	32 (20.3%)	1.000
Gestational age (w)	39.7 (38.9–40.1)	39.4 (38.7–40.3)	0.642
Total duration of labor (min)	725 (563–928)	748 (613–947)	0.575
Duration of labor analgesia (min)	570 (443–779)	573(417–757)	0.621
Fetal weight (g)	3287.8 ± 346.3	3307.3 ± 347.1	0.618

BMI, body mass index; GDM, gestational diabetes.

### 3.2 Postpartum neurological function at 14 days

On postpartum day 14, there were no significant differences between Group D and Group M in paresthesias (1.3% vs. 3.8%), decreased muscle strength (0.0% vs. 1.3%), and other neurological symptoms (0.6% vs. 0.0%) (*p* > 0.05) (Table 2).

**Table 2:** Comparison of neurological function between the two groups on postpartum day 14.

Variable	Group D (*n* = 157)	Group M (*n* = 158)	*χ* ^2^	*p-*value
Impaired skin sensation	6 (3.8%)	2 (1.3%)	2.026	0.283
Decreased muscle strength	2 (1.3%)	0 (0.0%)	2.000	0.498
Other neurological symptoms	0 (0.0%)	1 (0.6%)	1.010	0.498

All parturients with neurological symptoms at the 14-day follow-up had fully recovered in subsequent follow-up.

### 3.3 Clinical outcomes

Compared with Group M, Group D had a higher incidence of ERMF (7.6% vs. 2.5%) (*p* < 0.05). There were no significant differences in postpartum blood loss, nausea and vomiting, neonatal NICU admission rate, or instrumental delivery (*p* > 0.05) (Table 3).

**Table 3:** Comparison of clinical outcomes between the two groups.

Variable	Group D (*n* = 157)	Group M (*n* = 158)	*p-*value
Postpartum blood loss (mL)	280 (240–340)	260 (200–350)	0.089
ERMF	12 (7.6%)	4 (2.5%)	0.043
Nausea and vomiting	3 (1.9%)	8 (5.1%)	0.218
Instrumental delivery	9 (5.7%)	7 (4.4%)	0.619
NICU	26 (16.6%)	22 (13.9%)	0.534

ERMF, epidural-related maternal fever; NICU, Neonatal Intensive Care Unit.

### 3.4 Inflammatory markers

Compared with Group M, Group D had a significantly higher IL-6 level at 24 h postpartum (*p* < 0.001). IL-6 and CRP levels before labor analgesia, as well as CRP levels at 24 h postpartum, showed no significant differences (*p* > 0.05) (Table 4).

**Table 4:** Comparison of inflammatory markers between the two groups.

Variable	Group D (*n* = 157)	Group M (*n* = 158)	*p-*value
IL-6 (pg/ml)^a^	11.0 (8.9–13.9)	10.5 (8.5–12.7)	0.069
CRP (mg/L)^a^	5.9 (4.9–7.6)	6.2 (5.4–7.2)	0.559
24-h postpartum IL-6 (pg/mL)	26.1 (18.2–38.9)	15.4 (13.2–17.6)	<0.001
24-h postpartum CRP (mg/L)	47.9 (30.6–71.4)	49.7 (41.7–73.7)	0.061

a These variables were obtained before labor analgesia.

## 4 Discussion

This study compared the effects of dexamethasone versus methylprednisolone on neurological outcomes at 14 days postpartum in parturients who developed paresthesias during needle puncture or epidural catheter placement for combined spinal–epidural labor analgesia. Although methylprednisolone exhibited a trend toward fewer neurological events. The results showed no significant difference in neurological outcomes between the two groups, suggesting that, at the doses and timing used in this study, the two steroids were associated with similar neurological outcomes at 14 days postpartum. To our knowledge, this is the first randomized controlled trial to directly compare these two glucocorticoids on neurological outcomes following paresthesia during combined spinal-epidural labor analgesia. Although the primary outcome did not differ, the methylprednisolone group showed a trend toward lower early postpartum inflammatory levels, indicating a potential advantage in anti-inflammatory regulation.

The occurrence of paresthesia during puncture or catheter placement for labor analgesia indicates direct stimulation of the nerve roots, which may cause mechanical nerve injury—the extent of which is determined at the moment of insult. Glucocorticoids primarily exert their effects by alleviating secondary radicular edema and inflammatory responses, thereby creating favorable conditions for neurological recovery (15, 16). When the initial mechanical injury is mild, the subsequent inflammatory reaction is likewise modest; under such circumstances, either dexamethasone or methylprednisolone can effectively control local inflammation and promote neurological recovery, leading to similar recovery rates between the two groups. Furthermore, although the two steroids differ in chemical structure, pharmacokinetics, and distribution characteristics, the doses employed in this study have been shown to produce significant anti-inflammatory effects in the epidural space (17–19). Consequently, in the context of managing inflammation following minor puncture-related injury, the anti-inflammatory effects of the two agents may be comparable, masking potential differences in other pharmacological properties. Additionally, the primary outcome was assessed at 14 days postpartum, which represents a relatively short-term follow-up. For mild and transient nerve injuries, given that both interventions were effective, most patients had already recovered by this time point, making it more difficult to detect subtle between-group differences (20). Finally, the sample size, while sufficient for the primary power calculation, might still be limited for detecting statistically significant differences in low-incidence neurological outcomes, which should be considered when interpreting the non-significant trend. Thus, the absence of a significant difference between the two groups suggests comparable neurological outcomes following mild nerve injury, rather than demonstrating superiority of one intervention over the other.

Beyond the observed trend favoring methylprednisolone in neurological recovery, this study further demonstrated that parturients who received epidural dexamethasone had a significantly higher incidence of ERMF than those who received methylprednisolone (7.6% vs. 2.5%), along with higher serum IL-6 levels at 24 h postpartum. This suggests that dexamethasone may be less effective in suppressing systemic inflammatory responses. The difference may be related to the pharmacological properties of the two drugs: methylprednisolone has a shorter half-life and penetrates cell membranes more rapidly, allowing earlier and more potent suppression during the peak inflammatory phase after puncture injury (21, 22). In contrast, although dexamethasone is potent and has a long half-life, its onset of action is relatively slow, which may limit its immediate efficacy in controlling acute inflammatory bursts (23). This could explain the lower IL-6 levels and lower ERMF incidence in the methylprednisolone group. Elevated IL-6 is known to be closely associated with the development of ERMF, and ERMF itself has been linked to increased risks of adverse maternal and neonatal outcomes, such as cesarean delivery and NICU admission (24, 25). Therefore, methylprednisolone may offer superior protection against acute inflammatory responses.

This study has several limitations. First, it was conducted at a single center with a limited sample size, and the inclusion criteria were restricted to ASA II primiparous women with a BMI below 35 kg/m^2^, which may affect the generalizability of the results. Second, only parturients who experienced definite paresthesias during needle puncture or catheter placement were included; therefore, the findings may not apply to all individuals receiving labor analgesia. Third, fixed doses were used in both groups, and potential dose–response effects were not explored. Fourth, the assessment of the primary outcome at a single time point (14 days postpartum) is a significant limitation. This may have missed early neurological differences that resolved before day 14 and fails to capture long-term or permanent deficits. Given the low event rate, this endpoint likely reduces sensitivity to detect clinically meaningful differences between groups. Future studies should incorporate serial assessments with both early and extended follow-up. Fifth, the assessment of neurological outcomes via telephone interviews is a limitation, as it precludes objective evaluation of signs such as muscle strength. Sixth, the lack of a placebo control group is a limitation. Given that procedure-related paresthesias are often transient and may recover spontaneously, it remains uncertain whether the observed outcomes reflect drug efficacy or natural resolution. Therefore, this study cannot establish the superiority of corticosteroids over no intervention, and placebo-controlled trials are needed for further validation. Seventh, the very low incidence of persistent neurological events in both groups (≤3.8%) limited statistical power. Therefore, the non-significant findings reflect comparable short-term safety rather than equivalent preventive efficacy. Finally, inflammatory markers were measured at only a single time point, limiting insight into dynamic changes over time. Future studies with larger sample sizes are warranted to confirm these findings and to detect potential differences in low-frequency neurological outcomes.

## 5 Conclusion

In summary, among parturients who experienced paresthesia during combined spinal-epidural labor analgesia, epidural administration of dexamethasone or methylprednisolone led to comparable neurological outcomes at 14 days postpartum. Methylprednisolone demonstrated stronger anti-inflammatory effects, reflected by a significantly lower incidence of ERMF and lower postpartum IL-6 levels.

## Data Availability

The raw data supporting the conclusions of this article will be made available by the authors, without undue reservation.
